# Alteration of SHP-1/p-STAT3 Signaling: A Potential Target for Anticancer Therapy

**DOI:** 10.3390/ijms18061234

**Published:** 2017-06-08

**Authors:** Tzu-Ting Huang, Jung-Chen Su, Chun-Yu Liu, Chung-Wai Shiau, Kuen-Feng Chen

**Affiliations:** 1 Comprehensive Breast Health Center, Taipei Veterans General Hospital, No. 201, Sec. 2, Shih-Pai Road, Taipei 112, Taiwan; abby-eve@hotmail.com; 2 Division of Medical Oncology, Department of Oncology, Taipei Veterans General Hospital, No. 201, Sec. 2, Shih-Pai Road, Taipei 112, Taiwan; 3 Institute of Biopharmaceutical Sciences, National Yang-Ming University, No. 155, Sec. 2, Li-Nong Street, Taipei 112, Taiwan; jjjaannee@hotmail.com; 4 Faculty of Pharmacy, National Yang-Ming University, No. 155, Sec. 2, Li-Nong Street, Taipei 112, Taiwan; 5 School of Medicine, National Yang-Ming University, No. 155, Sec. 2, Li-Nong Street, Taipei 112, Taiwan; 6 Department of Medical Research, National Taiwan University Hospital, No. 7, Chung-Shan South Road, Taipei 100, Taiwan; 7 National Center of Excellence for Clinical Trial and Research, National Taiwan University Hospital, No. 7, Chung-Shan South Road, Taipei 100, Taiwan

**Keywords:** SHP-1 (SH2 domain-containing protein tyrosine phosphatase-1), STAT3 (signal transducer and activator of transcription 3), cancer therapy

## Abstract

The Src homology 2 (SH2) domain-containing protein tyrosine phosphatase 1 (SHP-1), a non-receptor protein tyrosine phosphatase, has been reported as a negative regulator of phosphorylated signal transducer and activator of transcription 3 (STAT3) and linked to tumor development. In this present review, we will discuss the importance and function of SHP-1/p-STAT3 signaling in nonmalignant conditions as well as malignancies, its cross-talk with other pathways, the current clinical development and the potential role of inhibitors of this pathway in anticancer therapy and clinical relevance of SHP-1/p-STAT3 in cancers. Lastly, we will summarize and highlight work involving novel drugs/compounds targeting SHP-1/p-STAT3 signaling and combined strategies that were/are discovered in our and our colleagues’ laboratories.

## 1. Introduction

Signal transducer and activator of transcription 3 (STAT3) is an oncogenic transcription factor which functions mainly through dimerization upon phosphorylation at tyrosine residues and translocation to cell nuclei [1,2]. STAT3 signaling is constitutively activated in various malignant human cancers and participating in multiple cellular progress as well as tumorigenesis [3,4]. Targeting STAT3 in cancer treatment has shown therapeutic benefits in both preclinical and clinical studies [5,6,7]. Common strategies for inhibiting STAT3 signaling includes inhibition of upstream receptors (Janus associated kinase (JAKs), interleukin-6 receptor), interfering STAT3 domain (block dimerization), impedance of STAT3-DNA binding, and inhibition of STAT3 transcription (by anti-sense oligonucleotides) and there have been corresponding investigational drugs/compounds to each strategy in various developmental stages [4,8,9,10,11]. Currently, no STAT3 inhibitors for cancer therapy have yet been approved, regardless of the mechanisms of STAT3 inhibition. There are several literatures that have nicely reviewed the progress of STAT3 inhibitors [10,11,12,13,14]. While many studies demonstrated that over-activation of Janus associated kinase (JAK) or growth factor receptor-associated tyrosine kinase (Src) contribute to the hyper-phosphorylation of STAT3 [15,16], phosphorylation of STAT3 is also tightly regulated by protein tyrosine phosphatases (PTPs) [17,18]. The Src homology 2 (SH2) domain-containing protein tyrosine phosphatase 1 (SHP-1), a non-receptor SH2 domain-containing PTP, has been reported to dephosphorylate STAT3 at its tyrosine 705 (Tyr 705) residue directly [19]. Recently, abnormal SHP-1/p-STAT3 signaling pathway was identified in various human malignant tumors, including multiple myeloma [20], hepatocellular carcinoma [21], breast cancer [22] and triple-negative breast cancer [23]. Therefore, targeting p-STAT3 by enhancing the activities of SHP-1 may be a strategy for inhibiting p-STAT3 in cancers. In this review, we detailed the recent knowledge of SHP-1/STAT3 signaling in cancer progression and potent anti-cancer agents that can target this pathway.

## 2. SHP-1/p-STAT3 Pathway in Cancers

Oncogenic STAT3 hyper-activation has been observed in various malignant human tumors, including lung, breast, colon, liver, prostate, stomach, pancreas, kidney and brain cancers [3]. The major phosphorylation sites in STAT3 are Tyr 705 and Ser 727. STAT3 activation induces the various cellular signaling required for cell survival (e.g., Bcl-xl, Bcl2, c-Myc, Mcl-1 and survivin), proliferation (e.g., cyclin D1), migration and invasion (e.g., MMP-2, MMP-7, MMP-9, Rho and Rac), as well as angiogenesis (e.g., vascular endothelial growth factor (VEGF) [4,11,24,25]. Besides its normal function, STAT3 has been shown to involve in the tumor development and cell transformation. Pioneering work indicates that constitutive STAT3 activation is found in v-Src-transformed cell lines [26] and is sufficient to induce transformation of normal breast or prostate epithelial cell lines and immortalized fibroblast [27]. These findings indicate that abnormal STAT3 expression can cause permanent changes in gene expression program and lead to a malignant phenotype.

To prevent inappropriately sustained STAT3 activation, there are multiple intrinsic regulators that can timely suppress STAT3 pathway. SHP-1 is one of PTP members that can accomplish this important function [28]. SHP-1 has been shown to dephosphorylate JAK kinases [29,30,31,32] and STAT3 directly [19] to silence the JAK/STAT pathway. SHP-1 is composed of three domains including N-SH2 domain, C-SH2 domain and catalytic PTP domain [33]. SHP-1 forms an auto-inhibitory conformation from N-SH2 domain to catalytic domain and maintains its enzyme activity in the inactive state [33,34]. SHP-1 has been shown to function as a tumor suppressor to inhibit the tumor growth including breast cancer [35], pancreatic cancer [36] and prostate cancer [37]. Previous studies have reported that SHP-1 is highly expressed in normal lymphoid cells, but diminished or abolished in many types of cancer cell lines and tumor tissues [38,39]. Growing evidence has revealed that loss of SHP-1 leads to constitutive activation of STAT3 in hematologic malignancies, since ectopic expression of SHP-1 in leukemia cells substantially decreases the levels of activated STAT3 [40]. Interestingly, STAT3 promotes epigenetic *SHP-1* gene silencing by forming complexes with DNA methyltransferase 1 (DNMT1) and histone deacetylase 1 (HDAC1) at the promoter region of SHP-1 [41]. Noteworthy, STAT3 activation usually accompanies high expression levels of DNMT1 to make an effective *SHP-1* gene silencing [41]. Taken together, loss of SHP-1 and sustained activated STAT3 activation may cause an oncogenic feedforward loop to render the malignant cells more sensitive to a series of extra- and intracellular stimuli.

The central link of STAT3 with other key oncogenic pathways such as activated protein 1 (AP-1) signaling complex, nuclear factor NF-κB and Wnt/β-catenin signaling makes STAT3 an attractive target and a master regulator for a plethora of cellular functions. For example, STAT3 regulates a broad range of transcription factors, such as c-fos, whereas c-fos is a key member of AP-1 proteins, a key cell life and death regulator [42]. The combination of STAT3 and AP-1 activities have been shown to drive elevated MMP-1 expression and promote colorectal cancer (CRC) invasion [43]. The cross-regulation between the Wnt/β-catenin and NF-κB signaling plays an important role in a diverse array of genes and pathways responsible for chronic inflammation, immunity, development, and tumorigenesis [44]. Aberrant activation and interaction of STAT3 and Wnt/β-catenin occurs in malignancies [45,46], and the convergence of these two pathways could regulate cell survival and stemness [47,48,49]. Moreover, NF-κB and STAT3 are crucial for controlling the abilities to resist apoptosis-based tumor surveillance as well as regulating angiogenesis and invasiveness in preneoplastic and malignant cells [50]. Notably, NF-κB and STAT3 can cooperate to promote cancer development and progression [51], and also regulate distinct functions in surrounding non-tumorigenic cells [52]. These key transcription factors NF-κB and AP-1 were also strongly activated in the absence of SHP-1 [53,54,55]. In addition, SHP-1 negative-regulated β-catenin transcriptional function and intestinal epithelial cell proliferation [56]. These studies further supported the cross-talk networks between the SHP-1/p-STAT3 pathway and these oncogenic signal transduction cascades. In addition to investigating the molecular mechanisms of cancer progression, these interactions also offer new insight into developing anti-cancer agents [51]. Future studies may help to delineate the impact of targeting SHP-1/STAT3 on the network among these pathways.

## 3. SHP-1/STAT3 Pathway Is a Target in the Treatment of Human Malignancies

SHP-1-mediated STAT3 downregulation is an appealing anti-cancer strategy to induce apoptosis in cancer cells. Previously, sorafenib has been demonstrated that can induce apoptosis in cancer cells through a novel kinase inhibition-independent mechanism. Sorafenib is the first [57] and is still the only Food and Drug Administration (FDA)-approved targeted therapy for advanced hepatocellular carcinoma cells (HCC) in 2016. Sorafenib has been reported to induce cell growth arrest and apoptosis in variety cancers including medulloblastomas [58], pancreatic cancer [59], glioblastoma [60], neuroblastoma [61], acute myeloid leukemia (AML) [62] and hepatocellular carcinoma (HCC) cells [63]. Our group has identified that sorafenib targets STAT3 in a kinase-independent pathway [19] and further generated a series of sorafenib derivatives (SC compounds such as SC-1, SC-40, SC-43, SC-49, SC-60 and SC-78) which lack activities on kinases but effectively induce cell apoptosis in cancers [19,64,65]. Sorafenib is a multiple kinase inhibitor targeting Raf-1 and other tyrosine kinases (e.g., VEGFR2, VEGFR3, Flt-3, PDGFR, and FGFR-1) [66,67]. In our works, sorafenib, but not its derivatives SC-1 [68], SC-43 [68] and SC-60 [69], significantly decrease the activity of Raf-1 kinase as well as the phosphorylation of VEGFR2 and PDGFRβ. We also proved that sorafenib and its analogues SC-1 and SC-43 showed no obvious effects on the phosphorylation of STAT3 upstream regulator JAK1 or JAK2, but effectively decreased the p-STAT3 proteins [68]. Sorafenib increased the enzyme activity of SHP-1 by directly interacting and impairing the association between the N-SH2 domain and the catalytic protein tyrosine phosphatase domain of SHP-1 [70]. We found that the N-terminal SH2 domain is a critical docking site of sorafenib [70]. Sorafenib derivatives SC-40 and SC-43, two potent SHP-1 enhancers, were also docked in the same site [70]. We therefore hypothesized that the interaction of sorafenib (or its derivatives SC-43 and SC-60) and the N-SH2 domain might lead to a release of the D61 catalytic site and activation of SHP-1 activity. Currently, the hypothesized mechanism was supported by using ectopic expressing dN1 (deleted N-SH2) and D61A mutant SHP-1 in cholangiocarcinoma [71], HCC [72], CRC [73], and triple-negative breast cancer (TNBC) [69] cells. Compared to wild-type SHP-1-expressing cells, SC-43 [71,73] and SC-60 [69,72] exerted less p-STAT3 downregulation and apoptosis-promoting effects on these mutant SHP-1-expressing cells. Compared with sorafenib, SC-1 and SC-43 induced more potent apoptosis in association with downregulation of p-STAT3 and its downstream molecules (cyclin D1 and survivin) in breast cancer cell lines [68]. The same mechanism existed in SC-60, a dimer-based sorafenib derivative that successfully exhibits anti-cancer ability in HCC [72] and TNBC [69]. Besides sorafenib, regorafenib, which is an inhibitor of multiple protein kinases, also exerts anti-tumor ability by reactivating SHP-1 and inhibiting STAT3 signaling in metastatic CRC [73,74] and HCC [75]. Su et al. also found that regorafenib and its analogue SC-78 inhibit cell growth and metastasis through SHP-1/p-STAT3/VEGF-A axis in TNBC [23]. Dovitinib, another multiple kinase inhibitor, induces significant apoptosis in HCC cells and sorafenib-resistant cells [76] by SHP-1-dependent STAT3 inhibition. SHP-1 has also been demonstrated as a target of nintedanib, which is a triple angiokinase inhibitor. Nintedanib induces HCC cell apoptosis by relieving the autoinhibitory structure of SHP-1 in an angiokinase-independent pathway [77]. Capillarisin which is derived from *Artemisia capillaris* induces SHP-1 to downregulate the expression of Jak1/2 and STAT3, and STAT3-regulated genes that mediate cell proliferation, cell survival, invasion, and angiogenesis [78].

**Figure 1:**
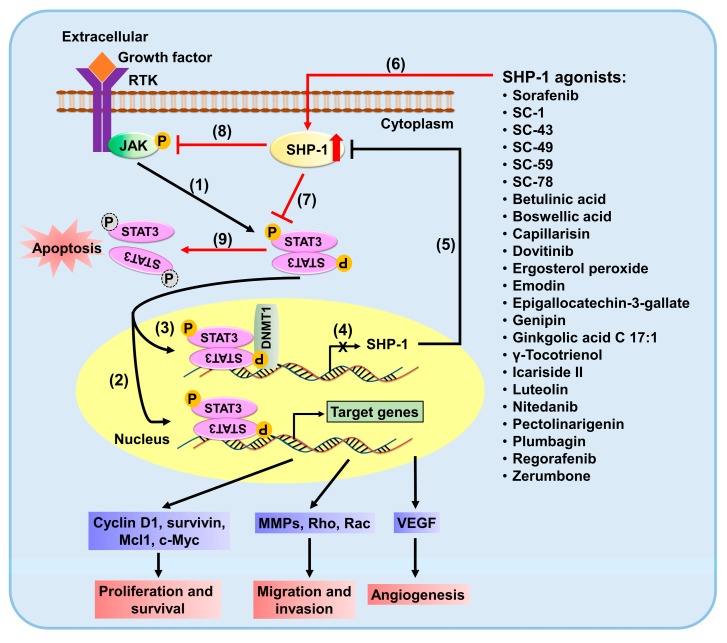
SH2 domain-containing protein tyrosine phosphatase 1 (SHP-1) inhibits signal transducer and activator of transcription 3 (STAT3) signaling pathways. In cancer cells, activated JAKs phosphorylates STAT3 (1), resulting in the translocation of activated STAT3 (p-STAT3) dimers to the nucleus as well as the activation of STAT3-regulated cellular proliferation and survival (cyclin D1, survivin, c-Myc and Mcl1), metastasis (MMPs, Rho and Rac) and angiogenesis (VEGFA) (2). Activated STAT3 also forms complexes with DNA methyltransferase 1 (DNMT1) at the promoter region of SHP-1 gene (3) to silence its transcription (4), leading to a decrement in protein levels (5). Enhancing SHP-1 activity by SHP-1 agonists (6) can directly dephosphorylate STAT3 (7) or its upstream JAKs (8) to decrease the p-STAT3 proteins (9) accompanied with the blockage of the STAT3-mediated cellular signaling pathways. Abbreviations: DNA methyltransferase 1 (DNMT1); Janus associated kinase (JAK); Matrix metalloproteinases (MMPs); Receptor tyrosine kinase (RTK); SHP-1 (SH2 domain-containing protein tyrosine phosphatase 1); Signal transducer and activator of transcription 3 (STAT3); Vascular endothelial growth factor (VEGF).

Apart from the above-mentioned drugs, there is a range of other anticancer compounds like Icariside II [79], betulinic acid [80], ergosterol peroxide [81], epigallocatechin-3-gallate [82], genipin [83], boswellic acid [84], ginkgolic acid C 17:1 [85], emodin [86], γ-Tocotrienol [87], honokiol [88], pectolinarigenin [89], and [90] zerumbone that modulates the expression or activity of SHP-1 to decrease the p-STAT3 proteins. Luteolin disrupts the interaction of HSP-90 and STAT3 and enhances SHP-1 to dephosphorylate STAT3 [91]. Hence, discovery and development of SHP-1 agonists may be a promising approach to combat tumor progression, drug resistance, and improve the survival rate as well as life quality of patients suffering from cancers. These findings suggest that SHP-1-mediated STAT3 downregulation is the potential target of anticancer drugs to inhibit cell growth and induce apoptosis in cancer cells. The regulation of SHP-1/p-STAT3 pathway and potent SHP-1 agonists are diagrammatically explained in Figure 1.

## 4. Selective Targeting SHP-1 Is Augmented by Combination Therapy with Chemotherapeutic Agents for STAT3 Signaling Blockade

Despite these promising results, SHP-1 agonist in combination with current approved chemotherapeutic agents may be synergistic to overcome toxicity, drug resistance or other side effects associated with high doses of single drugs. Sorafenib was the first and remains the only approved targeted therapy for advanced HCC in 2016. Comparison with single-agent sorafenib, sorafenib in combination with SC-43 had a synergistic effect on the increment of SHP-1 activity and the decrement of p-STAT3 in HCC cells [64]. In addition, combined sorafenib with SC-43 significantly promoted the apoptotic effect on sorafenib-resistant HCC cells [70]. Sorafenib and its derivative SC-49 also sensitized HCC cells to a novel anti-human death receptor 5 monoclonal antibody CS-1008-induced apoptosis through SHP-1-dependent STAT3 inhibition [92]. In radiation therapy for HCC, SC-59, which is another novel SHP-1 agonist, showed a better synergistic effect than sorafenib [93]. Combination therapy of sorafenib and SC-2001 (Mcl-1 inhibitor) inhibited STAT3 activation by RFX-dependent SHP-1 reactivation and defeated the sorafenib resistance in HCC and breast cancer cells [22,94,95]. Before the approval of sorafenib, the use of doxorubicin was common in advanced HCC treatment. Single-agent doxorubicin showed a 79% response rate in an initial phase II study [96]. However, subsequent studies exhibited limited efficacy and no obvious survival benefit [97,98,99,100,101]. Recently, potent SHP-1 agonists such as emodin [86], honokiol [88] and g-tocotrienol [87] effectively enhanced the apoptotic effect of doxorubicin and paclitaxel in HCC cells. γ-tocotrienol in combination with EGFR inhibitors (erlotinib or gefitinib) suppressed STAT3 and Akt signaling in murine mammary tumor cells [102]. Betulinic acid enhanced apoptosis induced by thalidomide and bortezomib in human multiple myeloma cells [80]. Dovitinib also acted as a novel radiosensitizer [103] and sensitized HCC cells to TNF-related apoptosis-inducing ligand (TRAIL) and tigatuzumab [104]. 5-azacytidine sensitized FLT3-ITD positive AML to lestaurtinib (CEP-701) by upregulating SHP-1 expression [105]. The combination of sorafenib and YC-1 (a soluble guanylyl cyclase activator) significantly displayed an anti-HCC effect compared with sorafenib or YC-1 used alone by modulating SHP-1/p-STAT3 pathway [106]. Plumbagin, a vitamin K3 analogue, has also been reported to inhibit STAT3 signaling by induction of SHP-1 and may have potential to sensitize STAT3-overexpressing cancers to thalidomide and bortezomib [107]. These studies provide the foundation for SHP-1/p-STAT3 modulators in conjunction with approved targeted agents in future cancer therapy.

## 5. Clinical Relevance of SHP-1/p-STAT3 in Cancers

Notwithstanding, the prognostic role of p-STAT3 on cancer patient outcome seems to be conflicting among various solid cancers [108]. Thomas et al. have summarized the studies on the relationship of JAK/STAT activation and prognosis [108]. In several cancers such as prostate, non-small cell lung cancers, cervical cancers, renal cell carcinoma (RCC) and glioblastoma, activation of STAT3 or STAT5 is associated with a worse prognosis; conversely, STAT3 is associated with favorable prognosis in breast cancer and in some studies in CRC and head and neck squamous cell carcinoma [108]. Different tumor biology among cancers, and the various regulatory mechanisms upstream of p-STAT3 signaling, for example, endogenous negative regulators such as the suppressor of cytokine signaling (SOCS) family, protein inhibitor of activated STAT (PIAS) proteins and the PTP family, or posttranslational modifications [109] may lead to the difference in prognosis prediction. STAT3 is highly expressed and activated in most breast cancers [110], especially in TNBC [111]. Also, high p-STAT3 levels correlated with worse outcomes in invasive breast cancers [112].

In contrast to the relatively established regulatory roles of SHP-1 in immune and hematopoietic cells [113,114], the clinical relevance of SHP-1 in cancers remain an exploratory field. There have been several studies on the expressions of SHP-1 and their significance in cancers. Some studies found decreased SHP-1 expression linked to poor outcome [115], whereas some concluded the opposite [116,117]. Immunohistochemical detection of SHP-1 has been shown to predict the outcome for localized prostate cancer; a decreased level of SHP-1 expression in prostate cancer cells is associated with a high proliferation rate and an increased risk of recurrence or clinical progression after radical prostatectomy for localized prostate cancer [115]. On the contrary, increased expression of SHP-1 is associated with local recurrence after radiotherapy for patients with nasopharyngeal carcinoma [117]. Insabato et al. found that elevated SHP-1 expressions were correlated with conventional pathologic parameters of tumor aggressiveness (such as HER2) and were associated with reduced patient survival [116]. Tao et al. found significant correlations among the infection of human papillomavirus (HPV) and the expression of SHP-1 in both condyloma acuminatum and cervical cancer, suggesting a putative role of SHP-1 in the progression of both condyloma acuminatum and cervical cancer after HPV infection [118]. Patients with an elevated SHP-1-E-cadherin axis had longer survival rate in CRC [119]. Given the relative paucity of literature on SHP-1 expressions in clinical cancer patient samples, more studies are needed to better define the prognostic roles of SHP-1 in various contexts of cancer types.

Currently, SC-compounds (SHP-1 agonists) are still in the pre-clinical stage, and none of these compounds have yet been approved as investigational new drugs (IND) for cancers. In contrast, STAT3 inhibitors that are designated based on more common strategies for inhibiting STAT3 transcriptional activity such as JAK inhibitors, STAT3 dimerization domain inhibitors, STAT3-DNA binding blockers, and antisense oligonucleotide inhibitor of STAT3 have been in various developmental stages or in clinical trials for cancer treatment (reviewed nicely in references [8,9,10,109]).

## 6. Conclusions and Perspectives

Loss of SHP-1 contributes to the activation of JAK/STAT3 as well as the other oncogenic pathways, and may further spark off an oncogenic feedforward loop to amplify tumorigenic signals. Hence, reactivating the function of SHP-1 phosphatase to target STAT3 can be a promising candidate for targeted cancer therapy and lay the foundation of new drug discovery. It is worth mentioning that SHP-1 has been reported as a potential target for cancer immunotherapy [120,121]. Loss of SHP-1 strongly increases the ability of Tregs to suppress inflammation [122]. In addition, blocking SHP-1 enzymatic activity by sodium stibogluconate significantly augmented the suppressor function of Treg both in vivo and ex vivo [122]. Furthermore, inhibition of SHP-1 has been shown to stabilize the conjugate formation between CD8 T-cells and antigen presenting cells [123], suggesting that SHP-1 blockage may lead to increased suppressor function. Although there is no report to elaborate the potential combination strategy of SHP-1 inhibition with other immune checkpoint inhibitors, it is still a promising and an important issue to investigate in the future.

The potential combination strategies of SHP-1 agonists and current approved chemotherapeutic drugs may also provide further insights into mechanisms of synergy and/or resistance, possibly leading to the development of clinical trials in human cancers. Analogously, abnormal SHP-1/p-STAT3 signaling can be a useful prognostic biomarker for predicting chemotherapeutic drugs’ response. These findings demonstrated an improved understanding of SHP-1/p-STAT3 interaction in promoting cancer progression and these concepts can be further translated into efficacious combination approaches to prolong the survival rate and improve the quality of life in patients. Future studies may help to delineate the impact of targeting SHP-1/STAT3 on the network among these pathways.
